# Nearly one out of every five adult TB patients suffered from food insecurity in Grawa District, Eastern Ethiopia: a multicenter facility-based cross-sectional study

**DOI:** 10.3389/fpubh.2023.1177618

**Published:** 2023-06-09

**Authors:** Jabir H. Abdulla, Berhe Gebremichael, Melat B. Maruta, Ibsa Yuye, Abdusalam Mohammed, Adera Debella, Ibsa Mussa

**Affiliations:** ^1^Department of Family Health, Grawa Health Bureau, Grawa, Ethiopia; ^2^School of Public Health, College of Health and Medical Sciences, Haramaya University, Harar, Ethiopia; ^3^School of Medicine, Obstetrics and Gynecology, Menelik Hospital, Addis Ababa, Ethiopia; ^4^Resource Mobilization and Health Financing, East Hararghe Zonal Health Office, Harar, Ethiopia; ^5^Revenue Authority, Dire Dawa City Administration, Dire Dawa, Ethiopia; ^6^School of Nursing and Midwifery, College of Health and Medical Sciences, Haramaya University, Harar, Ethiopia

**Keywords:** prevalence, food insecurity, tuberculosis, associated factors, Ethiopia

## Abstract

**Background:**

Despite a dramatic decline in tuberculosis mortality over the past 10 years, tuberculosis is still the leading cause of death globally. In the last 2 years, tuberculosis has affected an estimated 10 million individuals, and 1.4 million people have died worldwide. In Ethiopia, the weight of the problem is less known in the study area. As a result, the purpose of this study was to assess food insecurity and associated factors among adult patients with tuberculosis attending public health facilities in Grawa district, Eastern Ethiopia.

**Methods:**

A multicenter facility-based cross-sectional study was conducted from 01 March to 31 March 2022, among 488 randomly selected adult tuberculosis patients on treatment follow-up at public health facilities in Grawa district, Eastern Ethiopia. Data were collected using a pretested structured questionnaire through a face-to-face interview and document review, entered into EpiData version 3.1, and analyzed using SPSS version 25. The prevalence was reported using a 95% confidence interval (CI) and summary measures. Predictors were assessed using a multivariable logistic regression analysis model and reported using an adjusted odds ratio (AOR) with 95% CI. Statistical significance was declared at a *p*-value of <0.05.

**Results:**

Overall, the prevalence of food insecurity among the study participants was 19.5%, with a 95% CI (15.8%, 23.2%). Factors such as being male [AOR = 0.58, 95% CI: (0.34, 0.97)], being married [AOR = 2.93, 95% CI: (1.33, 6.47)], being merchant [AOR = 0.22, 95% CI: (0.04, 0.67)], having low wealth quintiles [AOR = 2.10,95%CI:(1.04–4.23)], receiving anti-TB treatment for two or fewer months [AOR = 0.48, 95% CI: (0.26–0.91)], using khat [AOR = 2.18, 95% CI: (1.29, 3.70)], and owning livestock (AOR = 0.56, 95% CI: 0.29–0.94) were significantly associated with food insecurity.

**Conclusions:**

According to this study, nearly one out of every five adults TB patients is food insecure. Factors such as being male, being married, being merchant, having low wealth quintiles, receiving anti-TB treatment for two or less months, those who chew mKhat and having a livestock were significantly associated with food insecurity. As a result, all stakeholders and concerned entities should prioritize improving the livelihood of TB patients through social security system programs, which are critical to the success of TB control and prevention efforts.

## Introduction

Globally, 821 million people suffer from chronic food deprivation, and the situation has been worsening in most regions of the African continent (1). Insecurity is generally defined as a lack of regular access to enough safe and nutritious food for normal growth and development and an active and healthy life (1, 2). Food insecurity has a substantial impact on the health of populations. Meanwhile, TB continues to be a major cause of morbidity and mortality globally, and its association with access to food and nutrition has been acknowledged for a long time (3). Tuberculosis holds a prominent place in public health, in part because it is listed as among the top causes of premature death among the adult population (4). Tuberculosis is present in all countries and affects all age groups. However, it affects the world disproportionately, as the majority of TB cases and deaths occur in developing countries, where more than half of the cases are in economically productive age groups (5).

Despite a dramatic decline in tuberculosis mortality over the past 10 years, tuberculosis is still the leading cause of death globally. Malnutrition and tuberculosis are two examples of how food insecurity has an impact on mortality, treatment failure, and tuberculosis risk (6). In the last 2 years, tuberculosis has affected an estimated 10 million individuals who have contracted the disease, and 1.4 million people have died worldwide (7).

The majority of people in developing nations experience food insecurity and an unhealthy environment, which have an immediate impact on household food access, availability, and consumption (7). Inadequate food intake leads to poor nutritional status and impaired immune function. Food access, availability, and consumption in households are directly impacted by food insecurity and an unhealthy environment, which are prevalent among the majority of people in developing countries (8). Poor nutritional status and impaired immunity are affected by insufficient dietary consumption. It was detected that there is a vicious cycle between undernutrition and TB, according to which poor nutritional status increases the risk of tuberculosis (TB), which in turn can lead to undernutrition (9).

Almost half (1 million) of all TB patients worldwide are malnourished (10). The World Health Organization (WHO) recommends that all TB patients have a nutritional assessment and receive the proper nutritional therapy to reduce the incidence of undernutrition (11). A sufficient, wellbalanced diet is linked to better weight gain and quicker sputum conversion (12). Energy-dense supplements also increased lean body mass and athletic performance (13). Patients with co-infections displayed significant wasting due to these synergistic negative effects (14, 15). Moreover, behavioral factors are linked to undernutrition; for example, smoking increases the risk of undernutrition, which may be linked to decreased appetite and increased resting energy expenditure due to nicotine's effects on body metabolism (16).

On the other hand, undernutrition among TB patients was at risk for those with a low socioeconomic position, low educational status, being female, having a positive sputum smear, being unable to work functionally, and not receiving dietary counseling (17–19). Unexpectedly, TB is Ethiopia's third-leading cause of hospital fatalities and the eighth-leading cause of hospital admissions (20). Furthermore, a small number of studies revealed that a sizeable portion of TB patients are undernourished (21, 22). Poor anti-TB medication adherence has also been found to be a regular occurrence in patients who are malnourished, which may increase the chance of acquiring multi-drug resistance (MDR) type TB, one of the pressing public health issues currently facing Ethiopia and the world (11, 22).

A reduction in undernutrition in the general population could significantly lower the incidence of TB because it is a powerful predictor of active TB (23). Hence, enhancing TB patients' nutritional states is crucial to lowering the likelihood of comorbidities and accompanying mortality as well as adverse treatment outcomes. Moreover, studies demonstrating the scope and causes of undernutrition are crucial for improving early case identification and management, yet Ethiopian literature is scant. Even the already conducted studies have a more limited scope, which eventually reduces the generalizability of the conclusion (22, 24).

Ethiopia has a major share of the global incidence of TB and its related mortality and morbidity (25). With an estimated 219,186 new cases and 48,910 TB deaths, the country has been ranked among the top countries with a burden of tuberculosis (26). On the other hand, food insecurity emerges as a key problem and development challenge in Ethiopia, with the country being ranked among the top East African countries affected by hunger and undernourishment (21). Subsequently, the combined impacts of food insecurity and tuberculosis place further strain on already limited resources as affected individuals strive to meet the food demand.

Nowadays, any individual infected with TB in Ethiopia receives free treatment. As an integral part of TB care and control, the health sector should recognize and help address generalized malnutrition, food insecurity, and other socioeconomic determinants and consequences of TB (27). Understanding the level of food insecurity and its associated factors among adult TB patients is crucial in designing an appropriate intervention to address the bidirectional deteriorating effects. However, despite the high burden of TB and troubling food insecurity in Ethiopia, only a few studies have been conducted concerning food insecurity and associated risk factors among adult TB patients (24, 28). Moreover, these studies have been limited to other areas, with no consideration of eastern Ethiopia, which is overwhelmed by the recurrent impact of drought and pose the risk of food insecurity (11, 29, 30).

Despite the commitment of various stakeholders and the government, the prevalence and determinants of food security, as well as tuberculosis, which are thought to be the direct and underlying causes of undernutrition, continue to be a problem. Moreover, there are a few studies conducted in Ethiopia that pointed out that food insecurity among Tb patients is a major widespread problem (11, 21, 31). Furthermore, there is a paucity of documented evidence regarding the problems under study generally at the county level and particularly at the study area level, so the purpose of this study was to assess factors associated with food insecurity among adult tuberculosis patients attending public health facilities in Grawa district, Eastern Ethiopia.

## Methods and materials

### Study design, setting, and period

A multicenter facility-based cross-sectional study was conducted from 01 March to 31 March 2022, among 488 randomly selected adult tuberculosis patients on treatment follow-up at public health facilities in Grawa district, Eastern Ethiopia. Regarding the location, Grawa is located in Eastern Ethiopia, 580 km from Ethiopia's capital, Addis Ababa. According to projections from the 2007 national census, the district will have a total population of 349,543 (179,248 men and 170,295 women) by 2020. The district has 45 health posts, 9 health centers, and 1 general hospital. From those district health facilities, six health centers and one hospital provided tuberculosis laboratory services. We used the STROBE cross-sectional checklist when writing our report (32).

### Eligibility criteria

All adult tuberculosis patients who had follow-up treatment in Grawa district public health facilities during the study period were considered the source population, whereas all adult TB patients who had follow-up treatment in randomly selected public health facilities and were available during the data collection period were regarded as the study population. The study excluded adult tuberculosis patients who were critically ill and unable to provide the necessary information during data collection.

### Data collection methods

An interviewer-administered, pretested, structured questionnaire adapted from the Household Food Insecurity Access Scale (HFIAS) for the measurement of food access was developed by USAID (33) and related published literature that contextualized the study objectives (34). It contains socio-demographic and economic characteristics, household-related conditions, substance use-related behavior, healthcare services, and comorbidity-related characteristics. The data were collected by six BSc Nurse Health professionals who fluently speak Afaan Oromo (the local language). Supervision was conducted by the principal investigator and two public health experts familiar with the study setting. Data collectors and supervisors were trained for 2 days on ethics, tool sampling, and data collection procedures. The data collection process was supervised on a daily basis, and timely feedback was communicated to the data collectors. First, we translated it while keeping the purpose of the questionnaire and the intent of the questions in mind. It was conducted by group members who speak both languages fluently translate it. To ensure the translation's accuracy, the questionnaire was translated back into English by someone who had not seen the original version and was unfamiliar with the questionnaire's context. The back-translated version is then compared to the original, and any meaning differences are corrected. After that, to ensure a cross-validity, we tried to interview a set of respondents in English and another set in the local language such as Afaan Oromo and Amharic, and their answers were then compared to detect differences in understanding. Finally, pretesting was conducted to identify questions that are poorly understood, ambiguous, or elicit hostile or other undesirable responses. We attempted to conduct a pretest using the already-translated questionnaire. We tried to implement all the steps in pretesting such as obtaining an evaluation of a questionnaire and testing the revised questionnaire through its paces on friends, colleagues, and so on. Moreover, when choosing a tool, reliability and validity must be taken into account. The consistency with which an instrument produces the same results across multiple trials is referred to as its reliability. The degree to which an instrument measures what it was designed to measure is known as its validity. Statistically, we performed Cronbach's alpha, which is a measure used to assess the quality of our employed instruments. The result was 0.87, which was within acceptable ranges.

### Variables and their measurement

#### Food security

A situation in which all people at all times have physical, social, and economic access to sufficient, safe, and nutritious foods that meet their dietary needs and food preferences for an active and healthy life (35).

#### Food insecurity

The lack of regular access to enough safe and nutritious food for normal growth and development and active and healthy life. This may be due to the unavailability of food and/or lack of resources to obtain food (36).

#### Food secure

May report worrying or being anxious about household food supply, but only rarely. Otherwise, the household does not experience any other conditions of inadequate food access.

#### Current substance use

Taking any substance such as alcohol, hashish, shisha, tobacco, and khat in the last 1 month prior to the study.

#### Ever substance use

Taking any substance such as alcohol, hashish, shisha, smoking cigarettes, and khat at least once in lifetime.

### Bias

There were a number of biases involved while conducting this research, and the researchers took explicit measures to avoid them. One of the biases was social desirability, and to avoid it, the researchers paraphrase the questions in a way that was not socially desirable.

### Sample size determination and sampling procedures

The sampling size was computed using the single population proportion formula, considering the following assumptions: 28.0% as the prevalence of undernutrition among adults with TB in southwest Ethiopia (28), a 95% level of confidence, and a 5% margin of error. A 10% non-response rate was also added to get a minimum sample size of 488.

**Figure 1:**
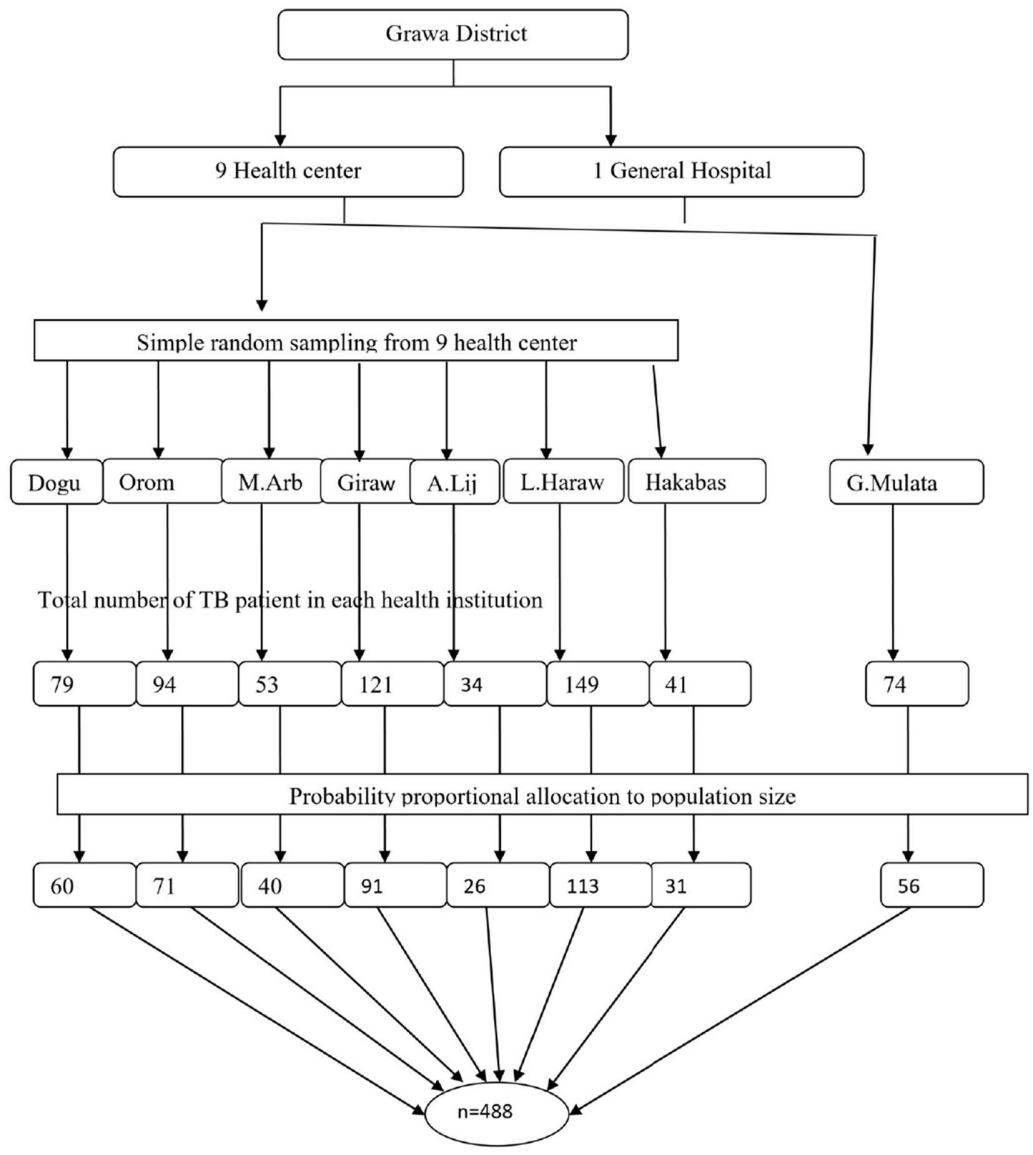
Schematic presentation of sample selection procedures of food insecurity among adult TB patients in Grawa District, Eastern Ethiopia.

To gather the study subjects, a multi-stage sampling technique was used. First, seven health centers from nine health centers in the Grawa district were selected by simple random sampling. Next, the total number of TB patients on follow-up treatment in each health institution was taken from the registration book of the patient to obtain a sampling frame (a list of eligible participants) before the actual data collection period. The probability-based proportional sampling technique was used to allocate the total sample size proportionally to each respective health institution. Then, each patient was given a unique code. Finally, study participants were selected using a simple random sampling technique (Figure 1).

### Data quality control

The questionnaire was initially prepared in English and then translated into the local languages by a bilingual expert (Afaan Oromo). Then, it was translated back into an English version to ensure its consistency. The data collectors and supervisor received training on the data collection tool and procedures. Before the actual study data collection, a pretest was conducted among 5% of the study participants in similar settings. The investigators and experienced research supervisors provided regular supervision.

### Data processing and analysis

First, the collected data were checked for completeness and consistency. Then, they were cleaned, coded, and entered into EpiData version 3.1 for further analysis. The entered data were exported to SPSS version 25 for analysis. Descriptive and summary statistics were conducted and reported using frequency tables and figures. A binary logistic regression model was fitted to check for an association between independent variables and the outcome variable. The model's fitness was checked by Hosmer–Lemeshow statistics and Omnibus tests. A multivariable analysis was performed to identify the true predictors of the outcome variables. A multi-collinearity test was carried out to check the presence of correlation between independent variables by using the standard error and co-linearity statistics, and no collinearity effects were detected. Thus, the value of the variance inflation factor (VIF) was 0.951. The direction and strength of the statistical association were measured by the odds ratio (OR) along with the 95% confidence interval (CI). A *p*-value of 0.05 was considered to be statistically significant in both bivariable and multivariable analyses.

## Results

### Socioeconomic and demographic characteristics

A total of 482 adult TB patients who were on treatment follow-up participated in the study, resulting in a response rate of 98.8%. The mean age of the study participants was 34.34 (SD ± 11.9) years, with 18 and 65 years being the minimum and maximum ages, respectively. Above half (53.5%) of the study participants were men. Most of the study participants 448 (92.9%) and 461 (95.6%) belonged to the Muslim religion and Oromo ethnicity, respectively. The majority, 411 (85.3%) of the participants, were rural dwellers. A total of 322 (62.2%) study participants had no formal education, and 311 (64.5%) of them were living with their spouses. Nearly a quarter, or 113 (23.4%), of the study participants were categorized as being in the high-wealth quintile (Table 1).

**Table 1:** Sociodemographic characteristics of adult TB patients undergoing treatment in public health facilities of Grawa District, Eastern Ethiopia, 2022 (*n* = 482).

**Variables**	**Categories**	**Frequency**	**Percentage**
**Age**	18–30	223	46.3
	31–45	177	36.7
	≥46	82	17.0
Sex	Male	257	53.3
	Female	225	53.3
Residence	Urban	71	14.7
	Rural	411	85.3
Ethnicity	Oromo	461	95.6
	Amhara	11	2.3
	Tigre	2	0.4
	Gurage	8	1.7
Religion	Muslim	448	92.9
	Chiristian	34	7.1
Education	No formal education	300	62.2
	Primary school	82	17
	Secondary school and above	100	20.7
Occupational status	Farmer	206	42.7
	Daily laborer	57	11.8
	Civil servant	12	2.5
	Merchant	38	7.9
	Others^*^	169	35.1
Wealth quintiles	Low	230	47.7
	Middle	150	31.1
	High	102	21.2

* Student and housewife.

**Figure 2:**
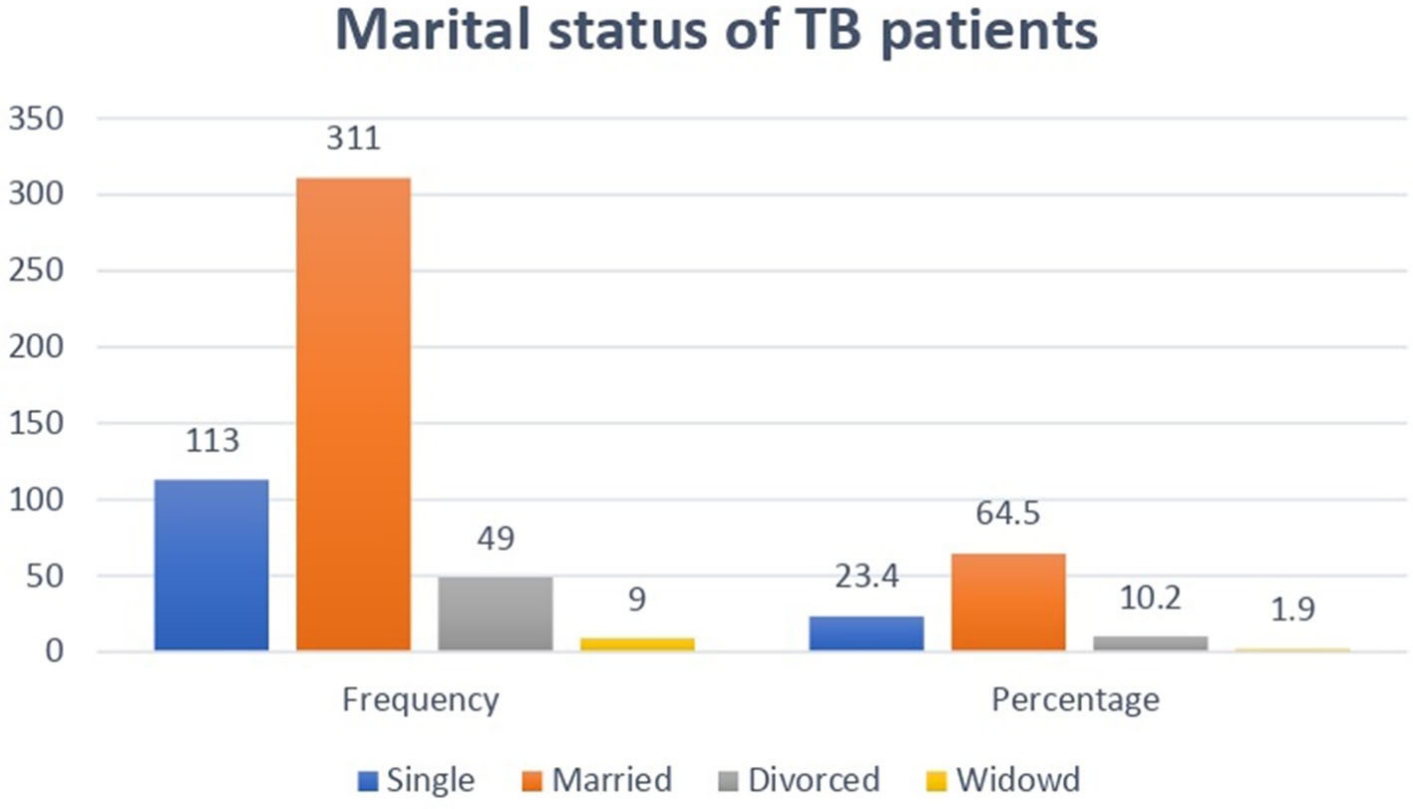
Distribution of marital status among adult TB patients undergoing treatment in public health facilities of Grawa District, Eastern Ethiopia, 2022.

As shown in Figure 2, among 482 study participants, 311 (64.5%) were married, and 9 (1.9%) were widowed.

### Household characteristics of the study participants

A total of 98 (20.3%) study subjects live alone in the household, whereas 308 (63.9%) study subjects live with four or more family members. The majority 322 (83.9%) of the households were monogamous. Nearly three-quarters 359 (74.5%) of the households had livestock, and 357 had farmland. Concerning food aid received from different organization, 22 (4.6%) households were beneficiaries of food support programs. Most of the 434 households (90%) have a latrine, and more than a quarter 134 (27.8%) of households have no window (Table 2).

**Table 2:** Household characteristics of adult TB patients undergoing treatment in public health facilities of Grawa District, Eastern Ethiopia, 2022 (*n* = 482).

**Variables**	**Categories**	**Frequency**	**Percentage**
Live alone in the household	Yes	98	20.3
	No	384	79.7
Number of people living in the household	Less than four	174	36.1
	Greater than or equal four	308	63.9
Have had employed family member	Yes	48	10
	No	434	90
Types of households (369)	Monogamous	307	< 83.2
	Polygamous	62	16.8
Household has farmland	Yes	357	74.1
	No	125	25.9
Household has livestock	Yes	359	74.5
	No	123	25.5
Household have latrine	Yes	434	90
	No	48	10
House has window	Yes	348	72.2
	No	134	27.8
Received food support from any organization	Yes	22	4.6
	No	460	95.6

### Health history of adult TB patients

More than three-quarters of the study subjects 371 (76.9%) were diagnosed with and treated for pulmonary tuberculosis, while the remaining 111 (23.1%) were diagnosed and treated for extrapulmonary tuberculosis. At the time of diagnosis, more than half of the cases 256 (53.2%) had a +1-sputum smear grade. In terms of treatment duration, 358 (74.3%) of patients received anti-TB treatment for more than 2 months, and 35 (7.3%) reported skipping anti-TB medication since starting treatment. The vast majority of respondents, 405 (84.1%), had no diagnosed comorbidity. Concerning the impact of health education on treatment adherence, the majority 401 (83.2%) of study subjects reported receiving health education from health professionals since enrolling in the treatment (Table 3).

**Table 3:** Health history of adult TB patients undergoing treatment in public health facilities of Grawa District, Eastern Ethiopia, 2022 (*n* = 482).

**Variables**	**Categories**	**Frequency**	**Percentage**
Type of TB	Pulmonary TB	371	76.9
	Extra pulmonary TB	111	23.1
Duration of anti-TB treatment	≤ 2 months	124	25.7
	>2 months	358	74.3
Sputum smear grading at diagnosis	+1	256	53.2
	+2	153	31.7
	+3	73	15.1
Have you experienced any other comorbidity in addition to TB?	Yes	77	15.9
	No	405	84.1
Did you delayed or skipped anti-TB drug?	Yes	35	7.3
	No	447	92.7
Received health education	Yes	401	83.2
	No	81	16.8

### Diet and lifestyle conditions of the study participants

Almost a quarter 118 (24.5%) of study participants reported eating fruit the week before the interview, while 152 (31.5%) reported eating vegetables. More than half of the respondents, 262 (54.4%), reported using khat, and 152 (31.3%) were smoking at the time of the treatment follow-up (Table 4).

**Table 4:** Diet and lifestyle conditions of adult TB patients undergoing treatment in public health facilities of Grawa District, Eastern Ethiopia, 2022 (*n* = 482).

**Variables**	**Categories**	**Frequency**	**Percentage**
Have you eaten vegetables in the last week?	Yes	152	31.5
	No	330	86.5
Have you eaten fruit in the last week	Yes	118	24.5
	No	364	75.5
Have you ever drunk alcohol?	Yes	17	3.5
	No	465	96.5
Have you ever chewed Khat?	Yes	262	54.4
	No	220	45.6
Did you smoke currently	Yes	151	31.3
	No	331	68.7
Received psychological support	Yes	141	29.3
	No	341	70.7

### Level of food insecurity

**Figure 3:**
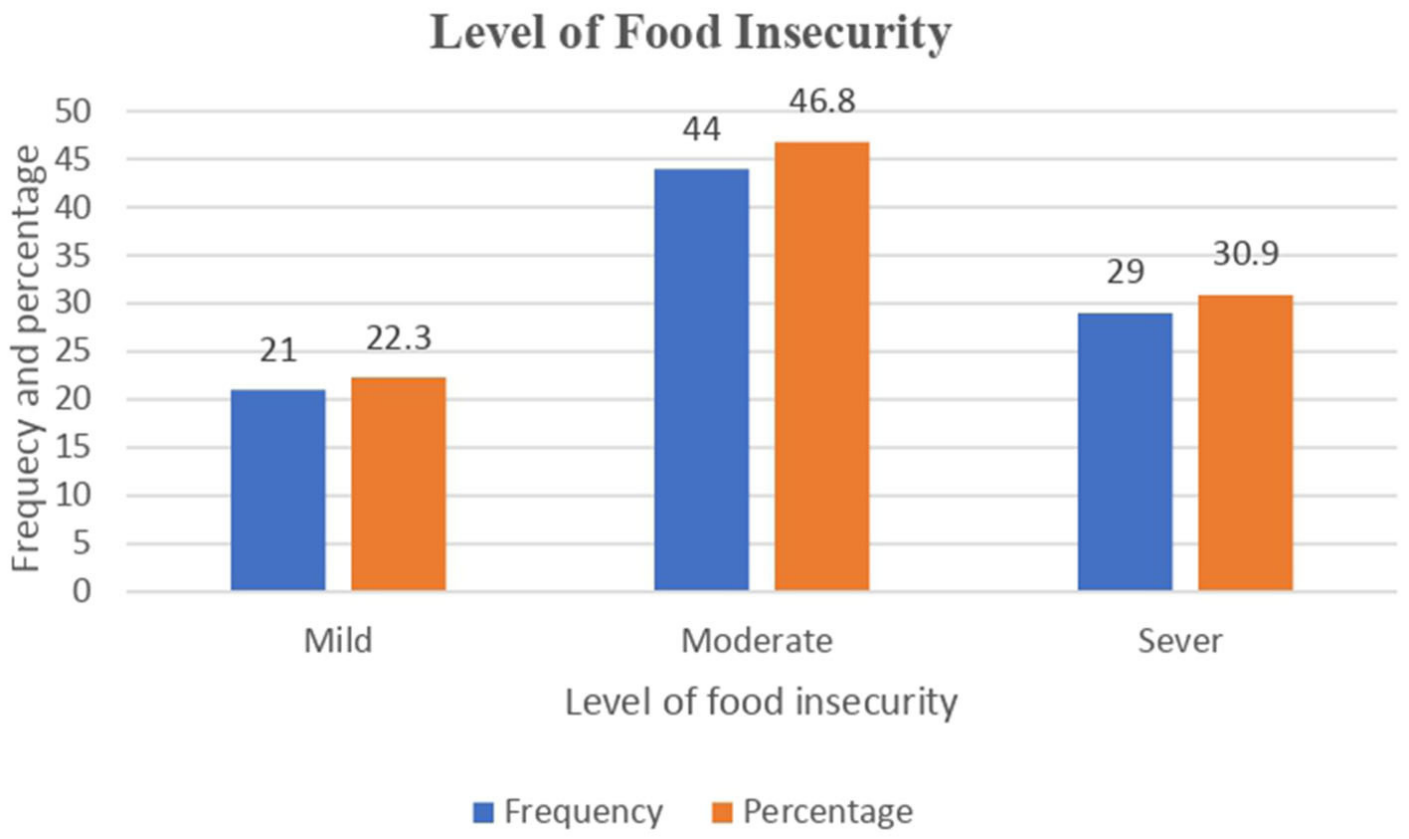
Level of food insecurity among adult TB patients undergoing treatment in public health facilities of Grawa District, Eastern Ethiopia, 2022.

According to the household food insecurity access scale, 94 patients were food insecure, with an overall proportion of 19.5% (95% CI: 15.8 to 23.2%). A total of 21 (22.3%), 44 (46.8%), and 29 (30.9%) TB patients with food insecurity had mild, moderate, and severe food insecurity, respectively. Nearly 72 (41.9%) patients reported being concerned about not having enough food to eat in the household, and 38 (7.9%) patients reported that household members went to bed hungry (Figure 3).

### Factors associated with food insecurity among the study participants

In the bivariable analysis, predictor variables such as the sex of the patient, marital status, wealth quintiles, being an urban resident, the khat chewing condition, duration of anti-TB treatment, having farmland, and the presence of livestock in the households were significantly associated with food insecurity among TB patients. However, in the final model of multivariable logistic regression analysis, predictor variables such as being male, being married, being a merchant, having low wealth quintiles, the duration of anti-TB treatment, chewing khat, and having livestock were factors that remained significantly associated with food insecurity.

Accordingly, male counterparts were 42% less likely to experience food insecurity as compared to their female counterparts [AOR = 0.58, 95% CI: 0.34–0.97]. Similarly, the likelihood of food insecurity was nearly three times higher among married TB patients (AOR = 2.93, 95% CI: 1.33–6.47). Furthermore, merchants were 78% less likely to experience food insecurity than their counterparts [AOR = 0.22, 95% CI: 0.04–0.67]. Likewise, the odds of having food insecurity were 2.10 times higher among TB patients who were in the lowest wealth quintile [AOR = 2.10, 95% CI: 1.04–4.23]. Furthermore, when compared to those who received anti-TB treatment for more than 2 months, those who received treatment for 2 months or less were 52% less likely to be food insecure [AOR = 0.48, 95% CI: 0.26, 0.91]. TB patients who chewed khat, on the other hand, were 2.18 times more likely to be food insecure than those who did not [AOR = 2.18, 95% CI: 1.29, 3.70]. Furthermore, TB patients with livestock in their households were 44% less likely to be food insecure than those without livestock [AOR = 0.56, 95% CI: 0.29, 0.94] (Table 5).

**Table 5:** Bi-variable and multivariable logistic regression analysis of factors associated with adult TB patients undergoing treatment in public health facilities of Grawa District, Eastern Ethiopia, 2022 (*n* = 482).

**Factors**	**Categories**	**Food insecurity**		**COR (95% CI)**	**AOR (95% CI)**
		**Yes (%)**	**No (%)**		
Sex of the patient	Male	39 (15.2)	218 (84.5)	0.55 (0.35–0.87)	0.58 (0.34–0.97) ^*^
	Female	55 (24.4)	170 (75.6)	1	1
Marital Status	Single	9 (8.0)	104 (92)	1	**1**
	Married	71 (22.8)	240 (77.2)	3.42 (1.65–7.10)	2.93 (1.33–6.47) ^*^
	Divorced	11 (22.4)	38 (77.6)	3.35 (1.29–8.70)	2.02 (0.69–5.89)
	Widowed	3 (33.3)	6 (66.7)	5.78 (1.23–27.06)	3.39 (0.59–19.66)
Residence	Urban	9 (12.7)	62 (87.3)	0.30 (0.12–0.75)^*^	0.56 (0.27–1.27)
	Rural	85 (20.7)	326 (79.3)	1	1
Religion of the patient	Muslim	83 (18.5)	365 (81.5)	0.48 (0.22–1.02)	0.62 (0.26–1.51)
	Christian	11 (32.4)	23 (67.6)	1	1
Educational status of the patient	No formal education	60 (20.0)	240 (80.0)	1	1
	Primary school	21 (25.6)	61 (74.4)	1.38 (0.78–2.44)	1.84 (0.95–3.58)
	Secondary and above	13 (13.0)	87 (87.0)	0.60 (0.31–1.14)	0.70 (0.32–1.53)
Occupation of the patient	Farmer	119 (57.8)	87 (42.2)	1	1
	Daily laborer	50 (88.1)	7 (11.9)	0.98 (0.48–2.01)	0.69 (0.25–1.91)
	Employee	6 (50)	6 (50)	0.34 (0.04–2.66)	0.26 (0.03–2.84)
	Merchant	7 (18)	31 (82)	0.59 (0.28–1.26)	0.22 (0.04–0.67)^*^
	Others^**^	103 (61)	66 (39)	0.64 (0.37–1.10)	0.73 (0.35–1.52)
Wealth quintiles	Low	55 (23.9)	175 (76.1)	1.98 (1.04–3.75)	2.10 (1.04–4.23) ^*^
	Middle	25 (16.7)	125 (83.3)	1.26 (0.61–2.55)	1.42 (0.66–3.03)
	High	14 (13.7)	88 (86.3)	1	1
Have had employed family member	Yes	16 (33.3)	32 (66.7)	2.28 (1.19–4.37)	1.85 (0.91–3.75)
	No	78 (18)	356 (82)	1	1
Duration of treatment follow up	≤ 2 months	15 (12.1)	109 (87.9)	0.49 (0.27, 0.88)	0.48 (0.26–0.91)^*^
	>2 months	79 (22.1)	279 (77.9)	1	1
Chewing Khat	Yes	63 (24)	199 (76)	1.93 (1.20–3.10)	2.18 (1.29–3.70)^*^
	No	31 (14.1)	189 (85.9)	1	1
Household has farmland	Yes	58 (16.2)	299 (83.8)	0.48 (0.30–0.77)	0.46 (0.24–1.09)
	No	36 (28.8)	89 (71.2)	0.48 (0.30–0.77)	0.46 (0.24–1.09)
Household have livestock	Yes	61 (17)	298 (83)	0.56 (0.34–0.91)	0.56 (0.29–0.94)^*^
	No	33 (26.8)	90 (73.2)	1	1

Key:

* *p*-value <0.01,

** *p*-value <0.001.

## Discussion

This study pointed out that 19.5% of the 95% CI (15.8%, 23.2%) study participants were found to be food insecure, of whom 22.3%, 46.8%, and 30.9% were mildly, moderately, and severely food insecure, respectively. Factors such as being male, being married, being a merchant, low wealth quintile, those who chew khat, duration of the anti-TB treatment, and presence of livestock in the households were identified as predictors of food insecurity.

In this study, nearly 19.5% of tuberculosis patients were food insecure. The findings from this study are in harmony with those of studies conducted in Vietnam (22%) (37) and South Africa (21%) (38). The similarities could be due to the fact that the two studies use a similar strategy for laboratory techniques and have a similar social structure. However, the current level of food insecurity is much higher than in previous studies conducted in different settings, like Sri Lanka (6%) (34). The discrepancy might be due to the variation in socioeconomic status and extended social security programs such as food safety nets among the study areas. In addition, seasonal variability when the studies were conducted could explain the observed difference among the findings. On the contrary, this finding was relatively lower than studies conducted elsewhere, such as in Indonesia (64%) (39), southwest Ethiopia (949.3%) (28), and south India (34.1%) (39). A possible justification could be the difference in the socioeconomic status among the study participants.

This study pointed out that being male was the strongest predictor of food insecurity among TB patients. Thus, male counterparts were 42% less likely to experience food security. This finding is supported by studies conducted in different settings, such as South Africa (38), the Somali Region (31), Adama (40), and low-income countries (41). The possible justification could be attributed to the fact that men have greater access to social capital and pathways out of crisis; for instance, their income pays off previous debts and secures new farm loans, whereas women frequently face severe time constraints due to their household food-security roles. Furthermore, gender roles may be a plausible explanation for this association. The majority of rural women in developing countries face financial and land-control constraints (41, 42). This may limit their ability to purchase high-demand food and reduce production, worsening food insecurity among them.

In the final model of multivariable analysis, marital status was found to be associated with food insecurity. Thus, those study participants who married were 2.93 times more likely to experience food insecurity as compared to their counterparts. Similar findings were reported from the studies conducted in Sri Lanka (34). The possible justification could be that a married couple has children who share a few resources, which absolutely plays a pivotal role in establishing and creating food insecurity in that specific household.

The current study found that low wealth quintile were another factor that was independently associated with food insecurity. Patients with tuberculosis in the lowest wealth quintile were more likely to be food insecure than those in the highest wealth quintile. This finding is in harmony with the study done in the Kembata Tembaro zone, southern Ethiopia (43), and south India (39), which found that TB patients with a low monthly income had twice the odds of experiencing household food insecurity as their high-income counterparts. This could be due to the fact that a low income limits an individual's purchasing power for food, potentially exacerbating food insecurity. Furthermore, catastrophic health expenditure as a result of tuberculosis diagnosis and treatment can exacerbate food insecurity in low-income groups throughout the disease's course (44). Similarly, a significant association was found between food insecurity and occupation in the current study, with merchants having lower odds of experiencing food insecurity than farmers. Thus, merchants were 78% less likely to be food insecure than their counterparts. This is in line with the findings of previous studies conducted in Nigeria and Ethiopia (45, 46). This could be due to merchants' purchase and access to power. Furthermore, they have a higher income than the others, allowing them to live a normal life free of food insecurity.

Furthermore, khat consumption was found to be significantly associated with food insecurity in this study. Patients who reported chewing khat had a higher risk of food insecurity than non-chewers. Several previous studies have found that food insecurity is significantly associated with substance use habits, which is consistent with the current findings (47–50). The current study's findings of increased food insecurity among khat chewers may be due to the negative effects of khat consumption on the household economy. Khat is a major cash crop and source of income for millions of households in eastern Ethiopia (51). It is frequently blamed for worsening food insecurity by diverting money to buy khat, complicating the chewer work culture (52), and displacing food crops (53). The current study's finding of an increased risk of food insecurity among khat chewers among TB patients may help researchers better understand how khat chewing affects the healthcare continuum in the study settings.

The current study also revealed that the duration of anti-TB treatment was significantly associated with food insecurity. Patients with TB who received anti-TB treatment for 2 months or less had lower odds of experiencing food insecurity compared with those who received anti-TB treatment for more than months. In contrast to the current finding, the study conducted in Burkina Faso reported no significant association between the duration of anti-TB treatment and undernutrition (54). A possible explanation for the association in the present findings could be from the perspective of the “health shocks notion,” where being on treatment for a longer duration resulted in extra expenditure incurred to receive healthcare and a loss in working days, which in turn adversely affected household earnings and food security (55, 56).

Finally, this study showed that study participants who had livestock were 44% less likely to experience food insecurity as compared to their counterparts. This is supported by the studies conducted in Ghana (57). This is because livestock is critical to food systems facing these emerging global challenges. Smallholders rely on livestock for income and labor-saving, productive assets. Livestock also helps with nutrition because animal-based foods are essential, especially in reducing child stunting in developing countries (58).

### Implications and limitations of study

This study plays a pivotal role in clearly showing the prevalence of food security among immune-compromised individuals such as TB patients. It also points out the factors that influence the problems under study. In this study, due to the nature of the study design, it would be impossible to determine the causal relationship between the variable and the outcome in the analysis. Moreover, the study failed to consider seasonal variability in effect which could bias the level of food insecurity.

## Conclusion

According to this study, nearly one out of every five adults TB patients is food insecure. Factors such as being male, being married, being merchant, having low wealth quintiles, receiving anti-TB treatment for two or less months, those who chew mKhat and having a livestock were significantly associated with food insecurity. As a result, all stakeholders and concerned entities should prioritize improving the livelihood of TB patients through social security system programs, which are critical to the success of TB control and prevention efforts.

## Data availability statement

The datasets used for this study are available from the corresponding authors upon reasonable request.

## Ethics statement

Ethical approval was obtained from the Institutional Health Research Ethics Review Committee (IHRERC) of Haramaya University, College of Health and Medical Sciences. Support letters from the College of Health and Medical Sciences were submitted to the selected health facilities where the study was conducted. After getting all permission letters from responsible bodies, informed, voluntary, written and signed consent was obtained from the study participants.

## Author contributions

All authors made a significant contribution to the work reported, whether that is in the conception, study design, execution, acquisition of data, analysis, and interpretation, or in all these areas, took part in drafting, revising, or critically reviewing the article, gave final approval of the version to be published, have agreed on the journal to which the article has been submitted, and agree to be accountable for all aspects of the work.
